# Ultrathin Metasurface-Enabled Electromagnetic Illusion for Target Platforms

**DOI:** 10.3390/mi17101146

**Published:** 2026-09-30

**Authors:** Wenlong Li, Jiangang Liang, Wenye Ji, Jiahe Wang, Guangzheng Ren, Yujie Hong, Jiajun Wu, Yali Zeng, Zhuochao Wang, Zhenxu Wang, Tong Cai

**Affiliations:** 1 Air and Missile Defense College, Air Force Engineering University, Xi’an 710051, China; wenll011204@163.com (W.L.); cat-liang1975@163.com (J.L.); jiwenyewave@163.com (W.J.); wangjiahe2025@163.com (J.W.); guangzheng_ren@163.com (G.R.); yujie__hong@163.com (Y.H.); 18049570445@163.com (J.W.); 2 School of Physical Science and Technology, Northwestern Polytechnical University, Xi’an 710129, China; yalizeng@nwpu.edu.cn; 3 Department of Chemistry, National University of Singapore, Singapore 117543, Singapore; zhuochao.wang@nus.edu.sg

**Keywords:** electromagnetic illusion, metasurface, wavefront manipulation

## Abstract

Benefiting from the unprecedented wavefront manipulation capability of metamaterials and metasurfaces, electromagnetic illusion technology has enabled customized tailoring of scattering characteristics with high precision. However, most existing electromagnetic illusion schemes are restricted to generating spurious scattering fields from empty space, suffering from inherent limitations such as narrow operating bandwidth and single application scenario, which severely hinder their applicability and reliability in complex electromagnetic environments. To overcome these bottlenecks, we propose an ultrathin meta-illusion for a target platform, which can simultaneously conceal the scattering signature of a real object and replicate that of a desired virtual target via amplitude and phase control of a metasurface. As a proof of concept, we design and fabricate a meta-illusion device that mimics the electromagnetic response of a scaled-down car, and experimentally validate its performance in the microwave regime. Experimental results show that with the target object loaded on the device, the meta-illusion achieves the correlation coefficients of near-field distributions and far-field patterns better than 86% and 85%, respectively, verifying the high fidelity of the proposed illusion scheme. This work provides a promising pathway for advanced electromagnetic camouflage, intelligent illusion engineering, and multifunctional metasurface systems.

## 1. Introduction

Precise control of electromagnetic scattering lies at the core of fundamental electromagnetic science and photonics research, underpinning a broad spectrum of applications from imaging to electromagnetic system engineering. Electromagnetic illusion, a paradigmatic wave-manipulation technology, enables tailored reconstruction of scattered wavefronts via precise modulation of electromagnetic wave propagation. It alters the electromagnetic signature of a target to emulate the response of a pre-designed virtual object at the observation side, thereby serving as a powerful approach for electromagnetic deception and interference. Early theoretical frameworks rooted in transformation optics [1] have laid a rigorous mathematical foundation for such wavefront engineering, spurring a wealth of complementary implementation strategies ranging from diffusive field cloaking to transmission-phase manipulation, all of which have witnessed substantial advances over past decades. The advent of metamaterials and their two-dimensional counterparts, metasurfaces [2,3,4,5,6,7], has further elevated electromagnetic wavefront manipulation to an unprecedented level, permitting arbitrary and flexible control over the full set of wave parameters including phase, amplitude, and polarization [8,9,10,11,12]. Such versatile wave-control capabilities have enabled a wide range of applications, including anomalous refraction and reflection [13,14,15], engineered antenna radiation patterns [16,17], adaptive radiation-scattering integration [18,19,20], and dispersion-engineered wavefront shaping. In particular, they have unlocked transformative possibilities for electromagnetic illusion and cloaking engineering. Driven by the growing demand for multifunctional operation and adaptability in complex electromagnetic environments, continuous breakthroughs have been made in this field, such as, dual-band illusion devices [21], broadband terahertz cloaks [22], and flexible-material-based illusion technologies [23]. More notably, the incorporation of active tunable elements [24,25,26,27,28,29,30,31,32,33] has fostered a new generation of intelligent illusion and cloaking systems [34,35,36,37,38] with programmable and reconfigurable functionalities, alongside related smart electromagnetic devices [39,40,41,42,43,44,45]. Despite these remarkable advances, a critical research gap remains.

Natural mirages have long captivated human imagination as a paradigmatic example of spontaneous optical wave manipulation. Originating from the gradient distribution of atmospheric refractive index, light rays undergo continuous bending during propagation, which spatially displaces the optical image of distant scenes. Consequently, observers at specific viewing angles perceive virtual imagery that does not physically exist at its apparent location. In essence, this ubiquitous natural phenomenon achieves precise control over wave propagation trajectories, reconstructing perceived visual reality and altering the apparent electromagnetic identity of physical scenes at the perceptual level. However, translating this elegant wave-control principle into engineered electromagnetic illusion technologies still faces fundamental practical bottlenecks. Most mainstream illusion schemes to date are restricted to generating spurious scattering signatures from empty free space, in other words, creating virtual electromagnetic responses without a physical target. This single-function manipulation mode severely limits their application scenarios, and is often accompanied by inherently narrow operating bandwidths. More critically, existing designs fail to simultaneously satisfy the dual requirements of virtual signature rendering and real target scattering concealment. This deficiency greatly undermines their applicability and reliability in complex real-world electromagnetic environments, where target masking and virtual impersonation must be implemented in tandem. Accordingly, achieving simultaneous suppression of the intrinsic scattering signature of a real object and faithful reproduction of the prescribed scattering characteristics of a desired virtual object remains a key technical bottleneck for next-generation electromagnetic illusion systems.

**Figure 1:**
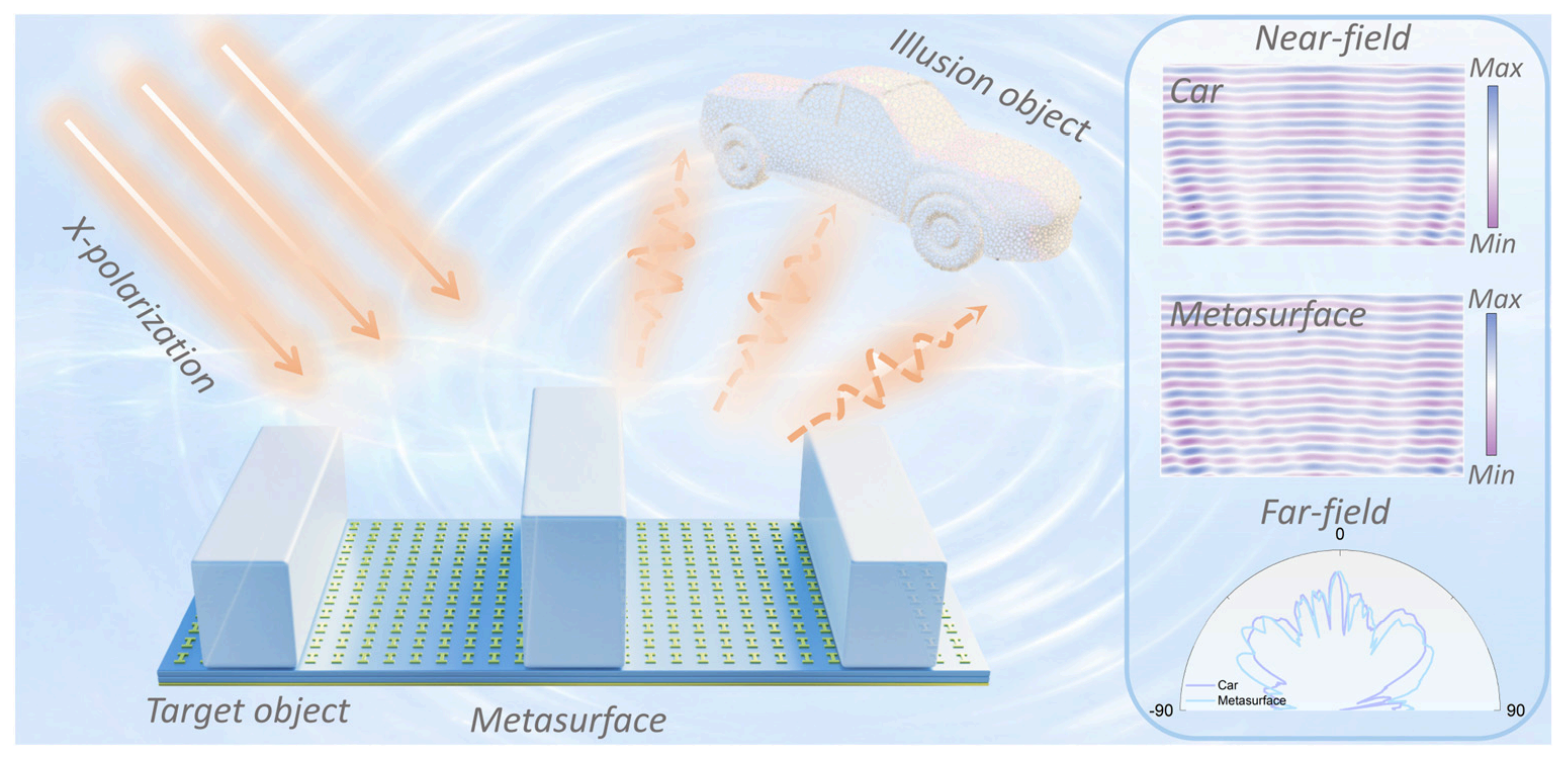
Conceptual schematic of the proposed ultrathin meta-illusion for a target platform. Under x-polarized electromagnetic wave illumination, the real target object to be disguised is placed directly on the elaborately designed metasurface: via precise wavefront engineering, the metasurface simultaneously suppresses the intrinsic scattering signature of the real target and replicates the electromagnetic response of a predefined virtual object (a scaled-down car), achieving high-fidelity electromagnetic identity replacement at the observation side. The right panel compares the near-field distributions and far-field scattering patterns of the virtual target and the meta-illusion device, intuitively demonstrating the high-fidelity matching performance of the proposed illusion scheme.

Drawing inspiration from the wave-manipulation principle inherent to natural mirages, we present an ultrathin meta-illusion for a target platform. As schematically illustrated in Figure 1, the proposed scheme integrates the object to be disguised directly onto an elaborately designed metasurface, which engineers both near-field distributions and far-field scattering patterns. In doing so, the meta-illusion device concurrently suppresses the intrinsic scattering signature of the real target and faithfully replicates the electromagnetic response of a predefined virtual object, achieving high-fidelity electromagnetic identity translation from the original object to the desired target at the detection side. As a proof-of-concept demonstration, we design and fabricate a meta-illusion prototype that transforms the scattering signature of a bulk test object into that of a scaled-down automobile model. Experimental characterizations conducted in a microwave anechoic chamber verify strong quantitative agreement between the proposed device and the target virtual object in both near-field mappings and far-field patterns, with correlation coefficients exceeding 86% and 85% respectively, fully validating its excellent illusion performance and broadband operational capability. This work addresses the limitations of conventional electromagnetic illusion technologies and provides a promising pathway toward future applications in electromagnetic camouflage, directional camouflage, and intelligent metasurface systems [46,47,48]. Furthermore, the proposed design methodology can be readily extended to higher frequency spectra, holding promising potential for applications in optical information processing and on-chip integrated photonic systems.

## 2. Results

The working principle of the proposed ultrathin meta-illusion for a target platform is schematically illustrated in Figure 1. The meta-illusion device is designed to satisfy the dual requirements of real target scattering concealment and virtual signature rendering, thereby demonstrating a mirage-inspired electromagnetic illusion for solid targets within the considered design framework. As a proof-of-concept demonstration, we select three cuboid objects as the real target to be disguised, which are placed directly atop the designed metasurface. By tailoring the phase response of each meta-atom via the proposed optimization algorithm, the scattered field of the loaded target is precisely engineered to match that of a predefined virtual object, here a scaled-down car model. The present demonstration focuses on these representative target configurations under x-polarized illumination, while further validation involving more diverse target geometries, heights, and virtual objects will be explored in future work. Under x-polarized wave illumination across the 19–23 GHz frequency band, the meta-illusion device produces near-field distributions and far-field scattering patterns highly consistent with those of the target car, while high-fidelity suppressing the original scattering features of the cuboid objects. In this manner, the functionality of concealing the real object and emulating a virtual one is achieved. The underlying phase compensation mechanism can be described as follows. For target objects with finite height such as the three cuboids, the meta-atoms directly beneath the objects compensate for the phase difference induced by the object’s vertical profile, yielding an equivalent response as if the target were placed on a flat reference metasurface. The required compensation phase is given by $\Delta \varphi =\pi +2k\left(f\left(x\right)-g\left(x\right)\right)cos\theta$, where ∆*φ* is the phase value to be compensated by the metasurface, k denotes the free-space wavenumber at the operating frequency, $f\left(x\right)\;$ and g(x) represent the surface profile functions of the target object and the metasurface, respectively, θ is the incident angle, and π is the phase shift introduced by the metallic medium. This phase compensation principle forms the theoretical foundation for the subsequent optimization algorithm. Notably, this analytical framework can in principle be extended to targets with different geometric profiles, indicating the potential scalability of the proposed design methodology.

**Figure 2:**
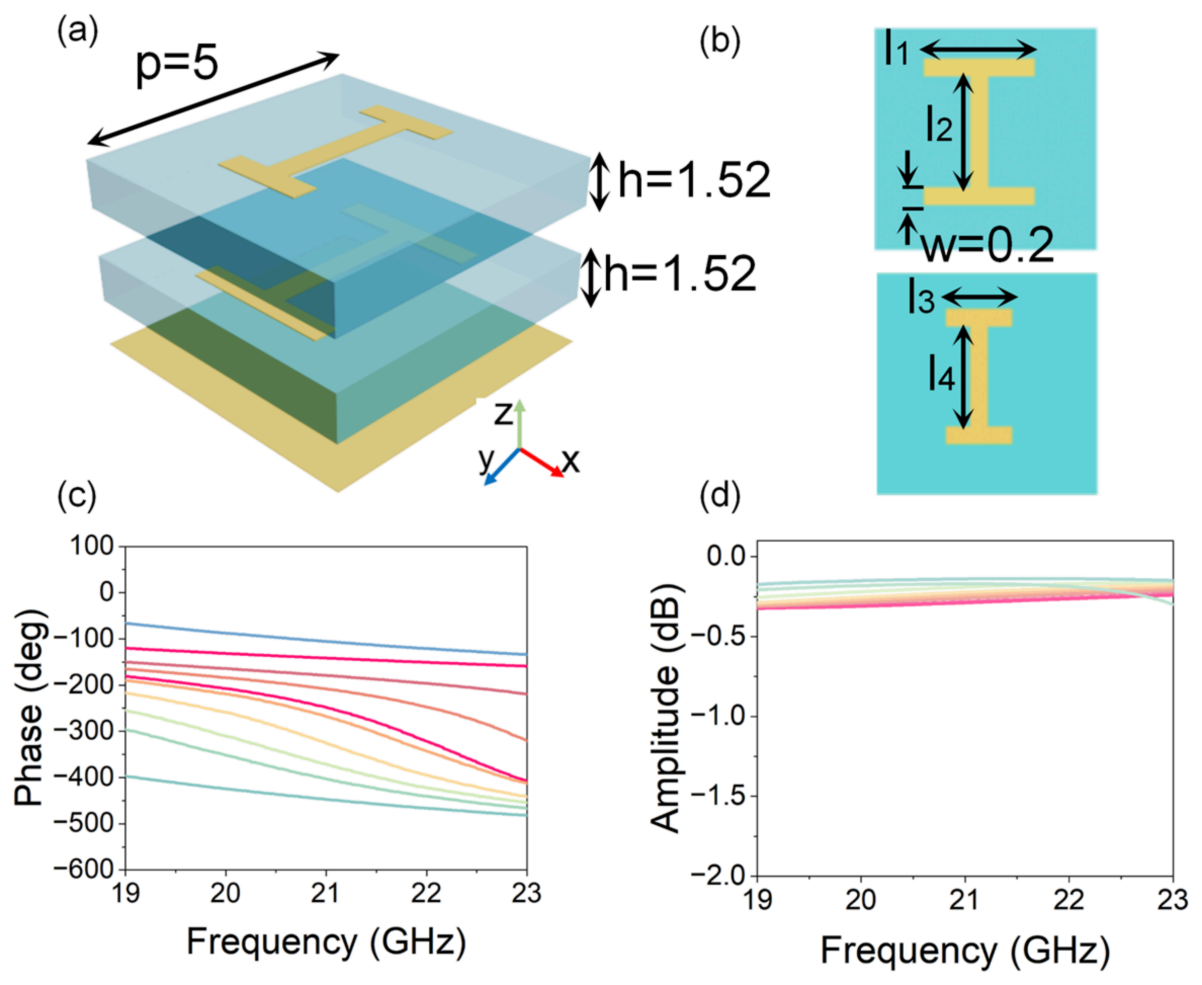
Design of the meta-atom. (**a**) Schematic of the overall structure of the meta-atom. (**b**) Exploded view of the bilayer structure. Varying the structural parameters of the meta-atom, the reflection phase (**c**) and the corresponding amplitude response (**d**) in the frequency range of 19–23 GHz.

To realize the above illusion functionality, the meta-atoms must provide full 360° phase coverage while maintaining high reflectivity across the operating band. The detailed meta-atom design is presented in Figure 2. Conventional single-layer meta-atoms benefit from fabrication simplicity but inherently suffer from limited phase coverage and narrow operational bandwidth, which would undermine the broadband performance of the illusion device. To overcome this limitation, we adopt a bilayer metallic structure to achieve nearly complete 360° phase control over a relatively broad frequency range. As depicted in Figure 2a,b, the meta-atom consists of three metallic layers separated by two dielectric spacer layers. Both the top and middle metallic layers adopt I-shaped patches, forming a cascaded reflective phase compensation architecture. By tuning the geometric dimensions of the I-shaped structures, flexible co-polarized reflection phase control is achieved under x-polarized incidence within 19–23 GHz. All dielectric spacer layers are fabricated from F4B substrate with a relative permittivity $\varepsilon_{r}=3.5$ and loss tangent tanδ=0.009. The meta-atom has a periodicity of p=5 mm, each dielectric layer has a thickness of h=1.52 mm, and the width of the metallic patches is w=0.2 mm. To characterize its phase coverage performance, full-wave numerical simulations are performed on the designed meta-atom. Figure 2c,d plot the simulated reflection responses as the structural parameters $l_{1},{\;l}_{2},{\;l}_{3}$, and $l_{4}$ are varied. Numerical results confirm that across the entire 19–23 GHz frequency band, the proposed meta-atom achieves full 360° phase coverage while retaining high co-polarized reflection efficiency, which fully meets the design requirements of the meta-illusion device.

**Figure 3:**
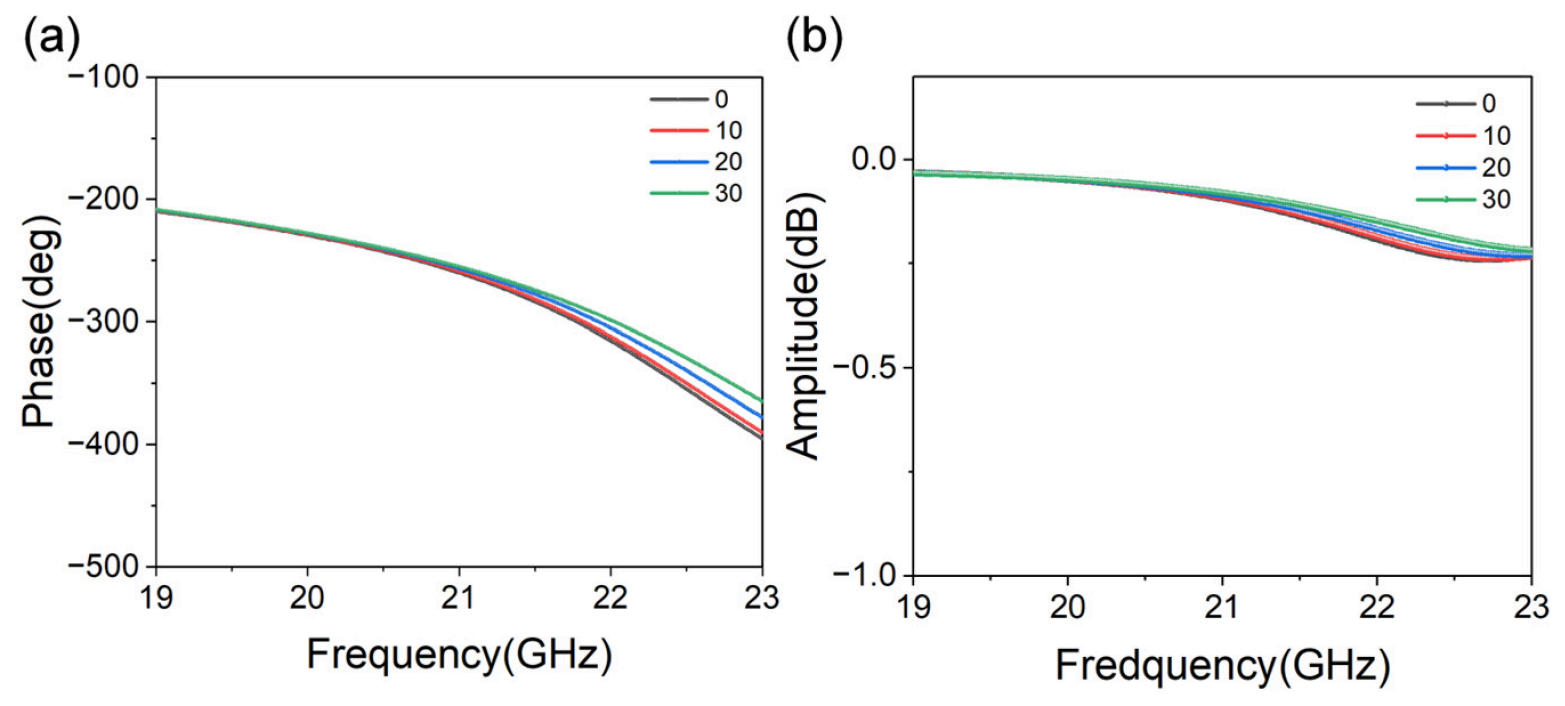
Angular response of the designed meta-atom under different incident angles. (**a**) Reflection phase responses and (**b**) corresponding reflection amplitude responses of the meta-atom under different incident angles.

Angular response of the designed meta-atom is further investigated to evaluate its robustness under oblique illumination. Since the proposed meta-illusion device is designed and experimentally demonstrated under normal x-polarized plane wave incidence, the angular stability of the meta-atom is mainly analyzed in terms of the reflection amplitude and phase response. As shown in Figure 3, the reflection characteristics of the meta-atom under different incident angles are simulated. With increasing incident angle, slight variations in resonance behavior can be observed, resulting in gradual phase deviation from the designed normal-incidence response. This phenomenon originates from the angle-dependent electromagnetic coupling between the incident wave and the multilayer metallic resonators. Nevertheless, within an incident angle range of 0–30°, the meta-atom maintains relatively high reflection efficiency and continuous phase coverage, indicating that the proposed design can tolerate moderate angular variations. It should be noted that the present meta-illusion scheme is optimized for normal x-polarized illumination, and the reported simulation and experimental results correspond to this specific operating condition. For applications requiring wider angular operation, further optimization strategies, such as angle-independent meta-atom design and multi-angle optimization, can be introduced to improve the angular robustness of the metasurface.

### 2.1. Working Principle

We first elaborate the design principle of the meta-illusion device from the perspective of evolutionary optimization. For the meta-illusion system where a real target is loaded on the metasurface under single-source illumination, the metasurface must simultaneously fulfill two phase compensation tasks, counteracting the phase perturbation induced by the intrinsic scattering of the real target, and imposing the phase profile required to replicate the scattering response of the target virtual car. The strong electromagnetic coupling between the target and the metasurface gives rise to a highly nonlinear inverse design problem, which cannot be readily solved via conventional analytical derivation. To address this challenge, evolutionary optimization algorithms (EOAs) are adopted in this work, which are renowned for their robust global search capability for complex nonlinear optimization objectives. As a well-established general-purpose optimization framework, EOAs exhibit excellent stability and adaptability. By mimicking population-based iterative evolution mechanisms, they enable efficient global exploration of complex solution spaces. Instead of modeling individual variables independently, EOAs encode the entire parameter space holistically, which significantly enhances their capacity to tackle high-dimensional design problems. For our meta-illusion design, the EOA framework can efficiently converge to the optimal phase configuration by iteratively evolving the meta-atom array, making it highly suitable for this nonlinear inverse design task. Specifically, the optimization objective of our meta-illusion for a target platform scheme is twofold, high-fidelity suppressing the intrinsic scattering signature of the real target, and faithfully reconstructing the near-field and far-field distributions of the desired virtual object. That is, the original scattering features of the real object are rendered undetectable at the observation side, while the target scattering profile is reproduced with high fidelity.

To establish the theoretical model, we take the predefined virtual car as an example and model it as a perfect electric conductor (PEC) protruding structure with arbitrary boundary profiles. The virtual object is discretized into a series of square grids, with a grid size matching the period of the metasurface unit cell, i.e., *p* mm. Under the ideal plane wave assumption, the wavefront phase evolves continuously as the electromagnetic wave propagates in free space. When the wavefront impinges on the surface of the virtual object, the incident phase at each discretized grid can be expressed as:

(1) $$\varphi_{\mathrm{inc}}=k_{0}\left(x_{m}\sin\theta_{i}\cos\varphi_{i}+y_{n}\sin\theta_{i}\sin\varphi_{i}+z_{k}\cos\theta_{i}\right)$$

where $x_{m},y_{n}$ denotes the in-plane coordinate of the grid indexed by (*m*, *n*) on the virtual object, and $z_{k}=h\left(x_{m},y_{n}\right)$ represents the corresponding height profile at this grid. θ*_i_* and φ*_i_* denote the elevation and azimuth angles of the incident source, respectively, and $k_{0}=2\pi /\lambda$ is the free-space wavenumber. From the height profile of each grid, the phase shift introduced by the surface height variation can be directly calculated, which is defined as the height-induced compensation phase $\varphi_{mod}=-\varphi_{mn}$. For far-field scattering calculation, the propagation phase from each grid to the far-field observation direction follows the far-field approximation, and can be written as:

(2) $$\varphi_{\mathrm{prop}}=k_{0}\left(x_{m}\sin\theta \cos\varphi +y_{n}\sin\theta \sin\varphi +z_{k}\cos\theta \right)$$

where (θ,φ) is the far-field observation angle from the grid indexed by (*m*, *n*). Under the far-field approximation, the scattered wave propagates as a plane wave, and the spatial amplitude decay caused by propagation distance is neglected. Combining the above phase components, the total far-field scattering pattern is calculated by coherent superposition of the contributions from all grids:

(3) $$E_{\mathrm{far}}\left(\theta ,\varphi \right)=\sum_{m=1}^{N_{x}}\sum_{n=1}^{N_{y}}A_{q}\cdot A_{mn}\cdot \exp\left[j\left(\varphi_{\mathrm{inc}}+\varphi_{\mathrm{mod}}-\varphi_{\mathrm{prop}}\right)\right]$$

where $A_{q}$ is the amplitude of the i-th incident source, and $A_{mn}$ is the reflection amplitude of the virtual object at grid (*m*, *n*). For simplicity, both quantities are normalized to unity in the following calculation.

For near-field calculation, the main difference from the far-field model lies in the spherical wave propagation nature. The definitions of incident phase and height compensation phase remain identical to those in the far-field model, while a spherical wave propagation model is adopted to describe the wave evolution from each unit cell to the near-field observation point. Here, the propagation distance R corresponds to the Euclidean distance from the unit-cell position to the observation point. Due to the divergence of the spherical wavefront, an amplitude attenuation factor 1/R is introduced into the calculation to account for the energy spreading effect. Accordingly, the near-field response at an arbitrary observation point can be formulated as:

(4) $$E_{\mathrm{near}}\left(S\right)=\sum_{m=1}^{N_{x}}\sum_{n=1}^{N_{y}}A_{q}\cdot A_{mn}\cdot \frac{\exp\left[j\left(\varphi_{\mathrm{in}}+\varphi_{\mathrm{mod}}-kR\right)\right]}{R}$$

where $R=\left\|S-S_{mn}\right\|$ is the distance from the unit-cell position to the observation point and $S_{mn}=(x_{m},\;y_{n},\;z_{k})$.

To improve the transparency of the optimization process, the principal optimization objective is briefly introduced here. The evolutionary optimization algorithm aims to minimize the discrepancy between the electromagnetic response generated by the optimized metasurface and the predefined virtual target. The fitness function can be expressed as:

(5) $$F(\phi )=\omega_{f}[\alpha S_{dB}+\beta S_{lin}+\gamma S_{radio}]+\omega_{n}S_{near}-P(\phi )$$

where ϕ represents the phase distribution of the metasurface, $S_{dB}$, $S_{lin}$, and $S_{radio}$ denote the far-field matching terms, $S_{near}$ represents the near-field similarity metric, and P(ϕ) is the phase constraint penalty term. The weighting coefficients are used to balance the contributions from far-field and near-field objectives. Detailed definitions of these terms and optimization parameters are provided in Supplementary Note S1.

**Figure 4:**
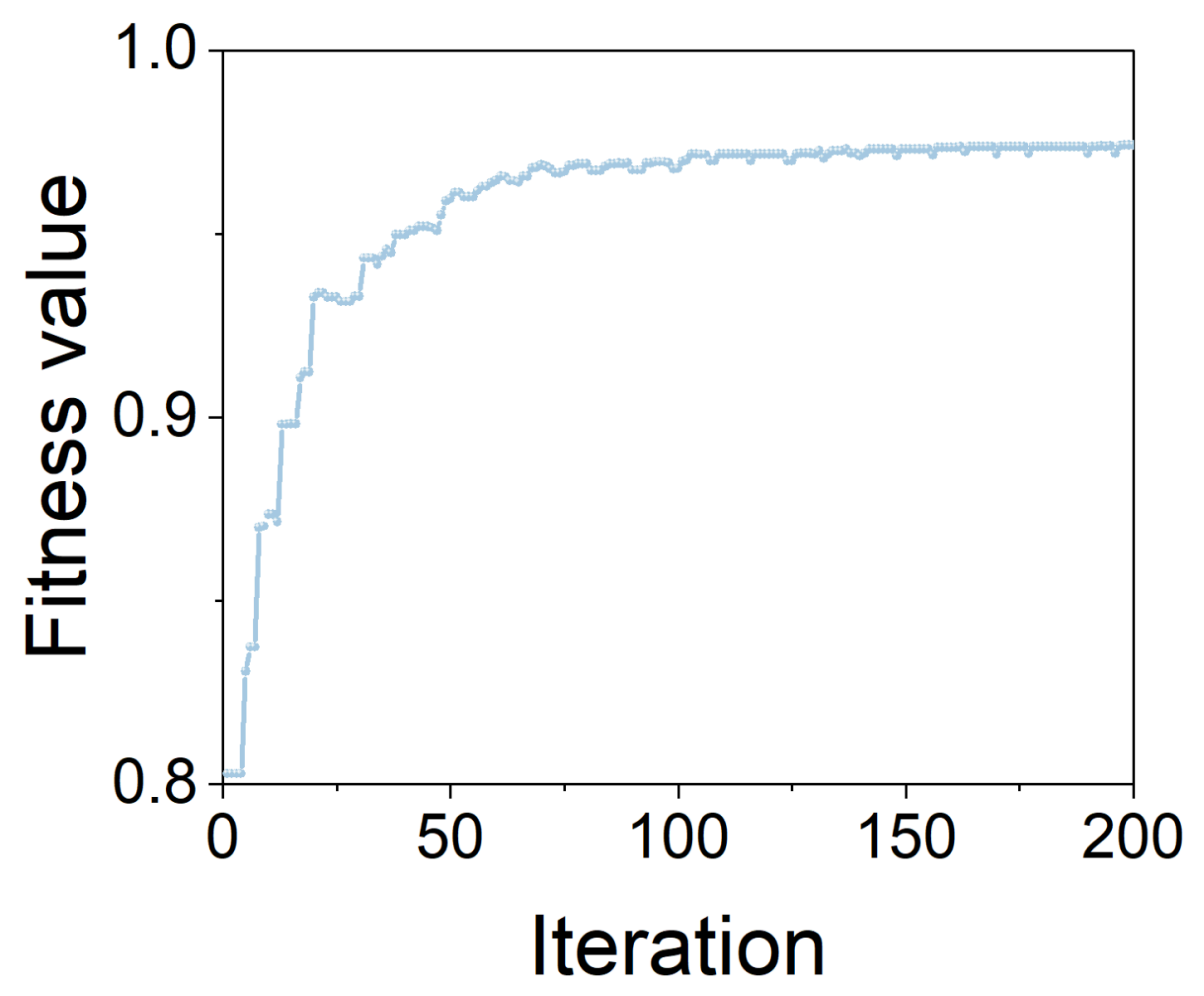
Convergence behavior of the evolutionary optimization algorithm. The fitness value as a function of iteration number demonstrates the progressive improvement in the matching performance between the optimized metasurface response and the predefined virtual target.

Furthermore, the convergence behavior of the evolutionary optimization process is analyzed. As shown in Figure 4, the fitness value gradually increases and approaches a stable state with increasing iterations, indicating that the optimization process progressively reduces the mismatch between the optimized metasurface response and the target electromagnetic signature. After sufficient iterations, the algorithm reaches a converged solution, providing the optimized phase distribution for the meta-illusion device. The detailed definition of the fitness function and the complete algorithmic flow are provided in the Supplementary Information.

In the scenario of ultrathin meta-illusion for a target platform, the optimization problem features a partially known solution, the phase configurations of meta-atoms directly beneath the real target are predetermined by the height compensation principle, while the phase profiles of the remaining meta-atoms are to be optimized via the EOA framework. Optimization results show that the correlation coefficients between the optimized scattered field and the target field exceed 0.9 for both far-field patterns and near-field distributions, verifying the effectiveness of the proposed design method.

### 2.2. The Meta-Illusion Design

**Figure 5:**
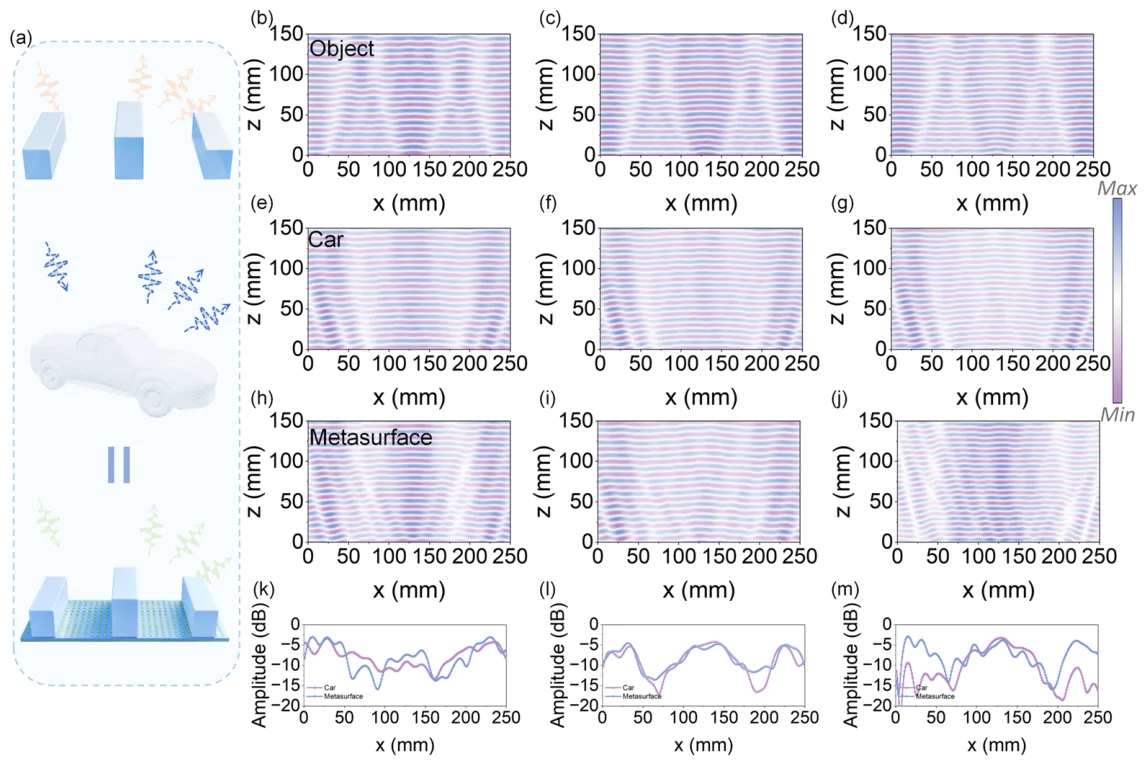
Full-wave simulated near-field performance of the meta-illusion. (**a**) Schematic illustration of the near-field verification principle. Distinct from the intrinsic scattering signature of the bare real target, the proposed meta-illusion device loaded with the identical target and optimized via the EOA generates a scattered field that closely replicates that of the predefined virtual car object. (**b**–**d**) Near-field electric field distributions of the standalone real target at 19, 21, and 23 GHz, respectively. (**e**–**g**) Corresponding near-field distributions of the reference virtual car object. (**h**–**j**) Near-field distributions of the meta-illusion device with the real target placed atop its surface. (**k**–**m**) Extracted one-dimensional electric field amplitude profiles along the x-axis at z = 80 mm, quantitatively comparing the response matching between the virtual target and the meta-illusion device at each operating frequency.

To numerically validate the feasibility of the proposed meta-illusion scheme, full-wave simulations are conducted using the commercial solver CST Microwave Studio. First, the three designed cuboid target objects are simulated as the baseline group. Their near-field scattering distributions at 19, 21, and 23 GHz are presented in Figure 5b–d. We then perform full-wave simulations for the reference virtual car to be emulated, with its corresponding near-field scattering profiles shown in Figure 5e–g. Direct comparison reveals pronounced discrepancies in scattered field patterns between the real cuboid targets and the virtual car, reflecting their fundamentally distinct intrinsic scattering signatures. Subsequently, we construct the complete metasurface array with phase configurations optimized via the EOA, and place the real target objects at the predefined positions on the metasurface. All simulations employ a time-domain solver under plane-wave excitation, with open boundary conditions enforced in all directions to replicate the free-space environment of practical microwave experiments. The resulting near-field distributions of the meta-illusion device are displayed in Figure 5h–j. Numerical results confirm that the proposed meta-illusion device effectively suppresses the intrinsic scattering signature of the real targets while faithfully reconstructing the desired scattering profile of the virtual object, successfully achieving the dual functionality of real target concealment and virtual scattering emulation.

To quantitatively evaluate the performance of the proposed illusion scheme, the correlation coefficient is adopted as the figure of merit, which characterizes the waveform matching degree between the scattered field distribution generated by the meta-illusion device and that of the reference virtual object. Mathematically, this coefficient is defined based on the cosine similarity metric, expressed as: $\frac{\sum_{i=1}^{n}\left(E_{i}^{meta}\times E_{i}^{obj}\right)}{\sqrt{\sum_{i=1}^{n}{\left(E_{i}^{meta}\right)}^{2}}\times \sqrt{\sum_{i=1}^{n}{\left(E_{i}^{obj}\right)}^{2}}}$, where $E_{i}^{meta}$ and $E_{i}^{obj}$ denote the electric field amplitudes at the i-th sampling point in the observation plane for the meta-illusion device and the reference virtual object, respectively. For a more intuitive quantitative comparison, one-dimensional electric field amplitude profiles extracted at the plane z=80 mm are presented in Figure 5k–m. The calculated correlation coefficients at 19, 21, and 23 GHz reach 0.94, 0.96, and 0.90, respectively. These high matching values verify that the proposed meta-illusion scheme achieves effective suppression of the real target’s intrinsic scattering while enabling high-fidelity reconstruction of the target near-field response under reflective operation.

**Figure 6:**
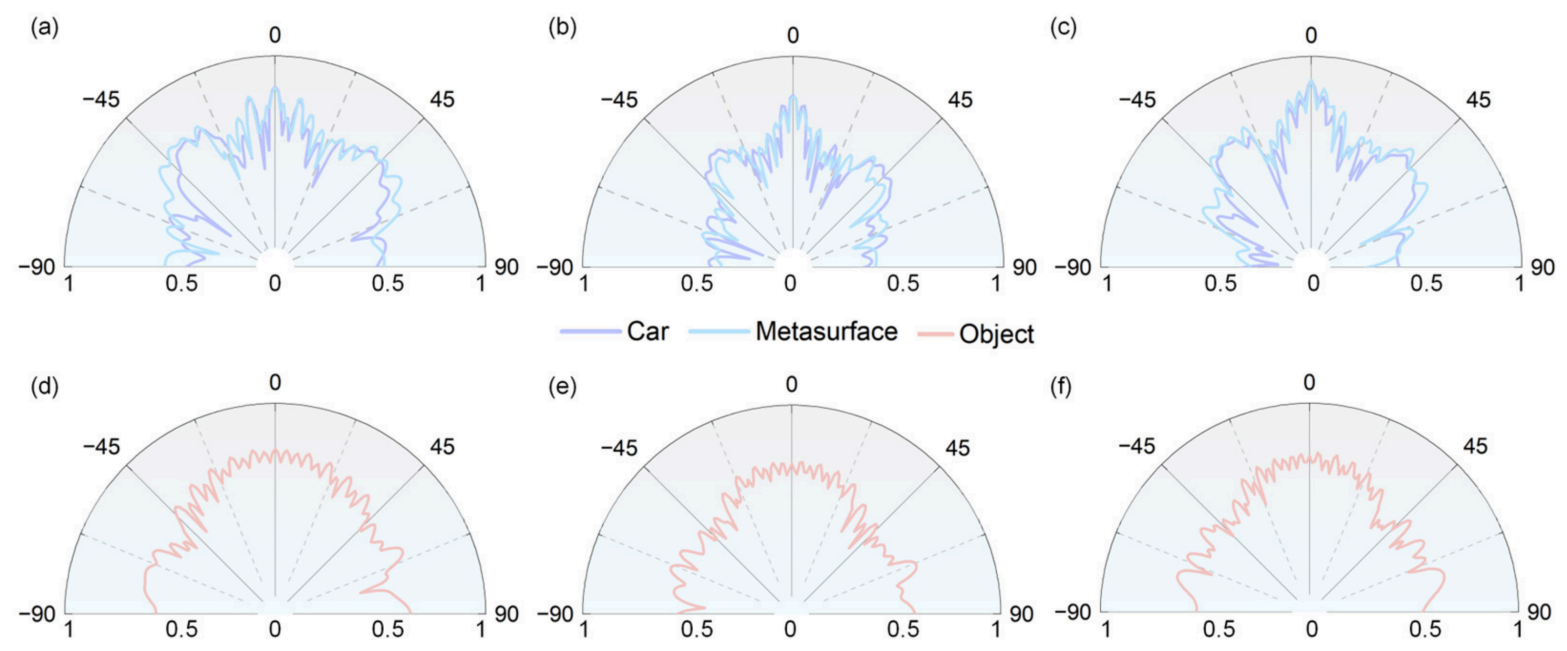
Full-wave simulated far-field scattering performance of the meta-illusion. (**a**–**c**) Comparative far-field scattering patterns of the meta-illusion device loaded with real cuboid targets and the reference virtual car at 19, 21, and 23 GHz, respectively. (**d**–**f**) Intrinsic far-field scattering patterns of the standalone real cuboid targets at the corresponding frequencies, serving as the baseline for performance comparison.

We further characterize the far-field scattering performance of the proposed meta-illusion device, with full-wave simulation results summarized in Figure 6. Figure 6d–f present the intrinsic far-field scattering patterns of the standalone cuboid targets at 19, 21, and 23 GHz, respectively, which serve as the baseline for evaluating scattering concealment performance. Figure 6a–c provide a direct comparison of far-field scattering profiles between the meta-illusion device and the reference virtual car across the same three operating frequencies. Quantitative calculation via cosine similarity yields correlation coefficients of 0.90, 0.95, and 0.94 at 19, 21, and 23 GHz, respectively. The high degree of waveform agreement verifies that the proposed meta-illusion achieves high-fidelity replication of the virtual target’s far-field scattering signature, while effectively suppressing the original scattering characteristics of the real target.

### 2.3. Experimental Realization of the Meta-Illusion

To further validate the practical performance of the proposed ultrathin meta-illusion for a target platform strategy beyond numerical simulations, we fabricate a meta-illusion prototype using an F4B dielectric substrate with a relative permittivity $\varepsilon_{r}=3.5$ and loss tangent tanδ=0.009. The prototype is manufactured via a standard printed circuit board (PCB) process, comprising a 50×20 meta-atom array with an overall physical dimension of 100 mm×260 mm. The scaled-down reference car model and the cuboid target objects are fabricated via 3D printing, and photographic images of all fabricated samples are provided in Figure S2 of the Supplementary Information. To quantitatively characterize the illusion fidelity, systematic near-field and far-field experimental measurements are conducted with the three cuboid targets placed atop the metasurface, to evaluate the scattering field correlation between the meta-illusion device and the physical reference object.

**Figure 7:**
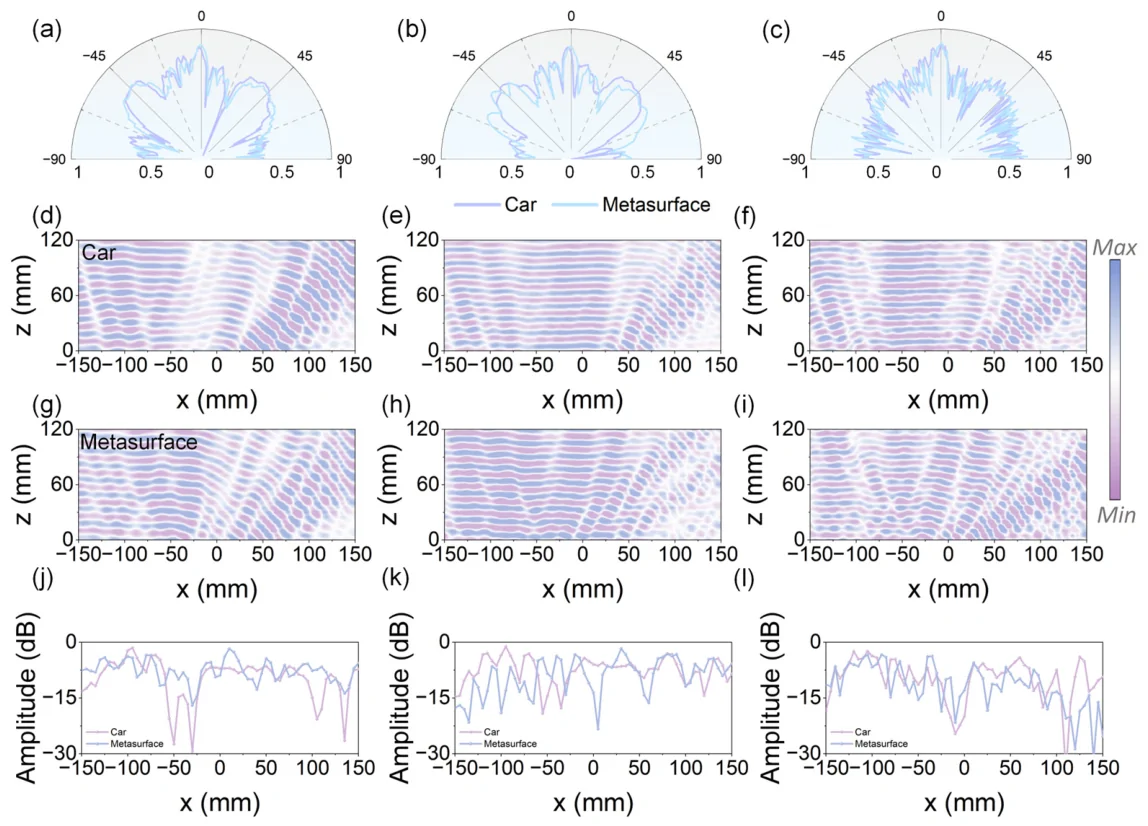
Experimental verification of the meta-illusion. (**a**–**c**) Comparative far-field scattering patterns between the meta-illusion device loaded with real cuboid targets and the reference virtual car at 19, 21, and 23 GHz, respectively. (**d**–**f**) Measured near-field electric field distributions of the reference virtual car at the three corresponding operating frequencies. (**g**–**i**) Measured near-field electric field distributions of the meta-illusion device with real cuboid targets placed atop its surface. (**j**–**l**) Extracted one-dimensional electric field amplitude profiles along the x-axis at z = 80 mm, for quantitative comparison of the field matching degree between the reference car and the meta-illusion device.

To experimentally validate the meta-illusion performance of the proposed meta-illusion device, we first carry out far-field scattering measurements, which directly characterize the far-field scattering signature that determines the external electromagnetic perception of the target. Both the transmitting and receiving horn antennas are connected to a vector network analyzer (VNA), which functions simultaneously as the microwave signal source and the receiver for scattered signals. The transmitting horn antenna and the meta-illusion sample are mounted together on a 360° rotary stage, enabling angle-resolved acquisition of the far-field scattering patterns. The measurement covers a broad frequency range of 18–26 GHz, fully encompassing the target operating band of the device. During the measurement, the receiving antenna remains fixed, while the rotary stage rotates stepwise to collect the scattered field amplitude at each observation angle. The transmitting antenna is placed approximately 2 m from the sample, and the receiving antenna is located 10 m away from the meta-illusion device, which fully satisfies the far-field condition across the entire operating frequency band. Detailed information about the near-field and far-field measurement configurations is provided in Supplementary Note S2. Measured far-field results are presented in Figure 7a–c. The far-field scattering patterns of the proposed meta-illusion device exhibit high overall consistency with those of the reference car target. At the three representative frequencies of 19, 21, and 23 GHz, the cosine similarity coefficients of the scattering profiles reach 0.88, 0.93, and 0.85, respectively. To further demonstrate the broadband performance, continuous frequency-sweep measurements are performed over the entire operating band of 19–23 GHz. The corresponding frequency-dependent correlation coefficients of the near-field and far-field responses are provided in Supplementary Figure S4. The results show that the correlation coefficients remain stable throughout the operating band, further validating the broadband electromagnetic illusion capability of the proposed device. These quantitative results corroborate that the proposed meta-illusion scheme can faithfully reconstruct the far-field scattering signature of the desired virtual target at the macroscopic observation level.

**Figure 8:**
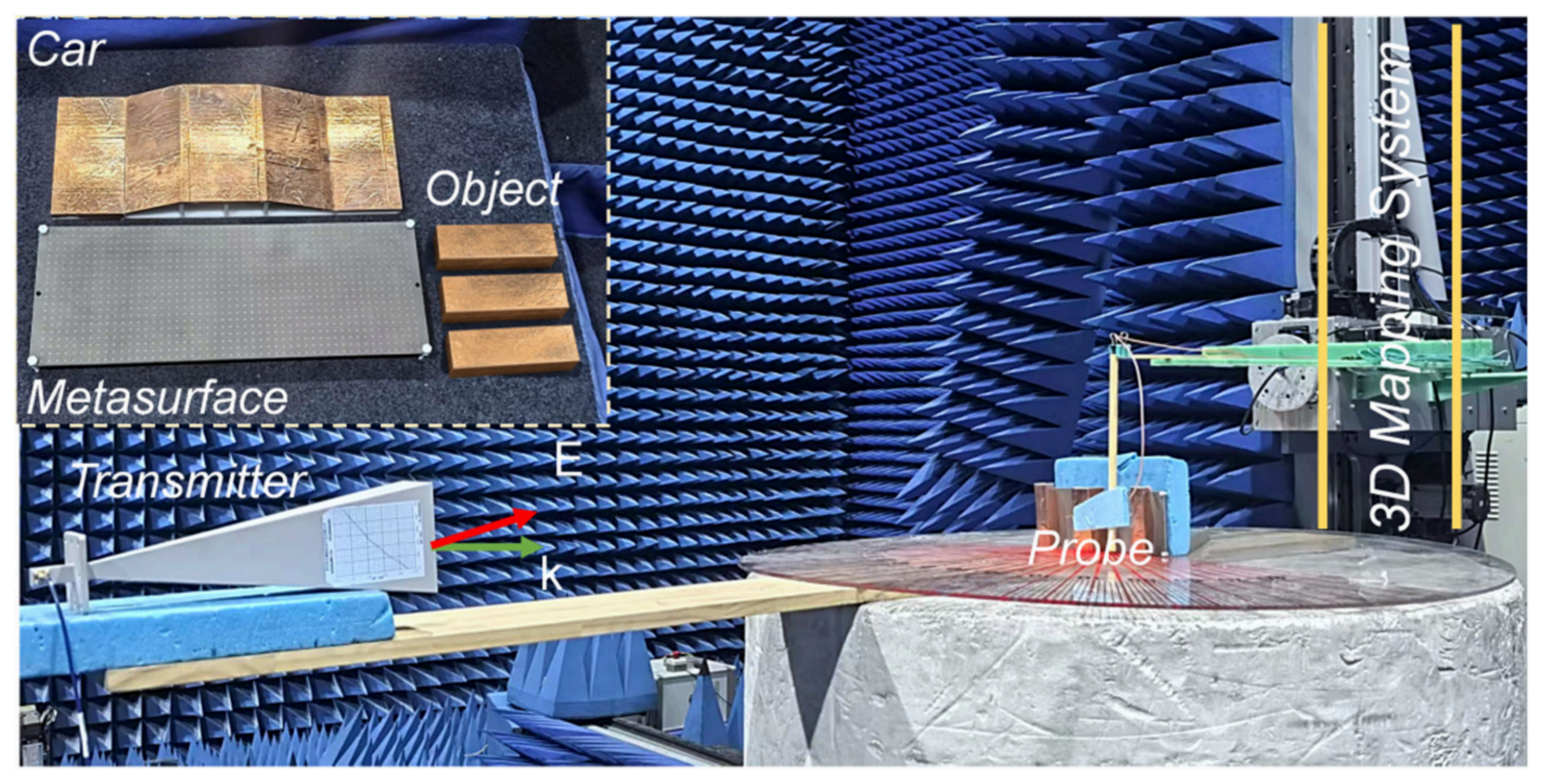
Near-field scanning measurement setup.

To further reveal the underlying wave-manipulation mechanism and verify the field matching performance at a finer spatial scale, we subsequently conduct systematic near-field scattering measurements on the fabricated prototype. As illustrates in Figure 8, a transmitting horn antenna and a near-field scanning probe are both connected to the VNA system. To suppress parasitic near-field coupling between the probe and the sample, the minimum distance between the scanning probe and the meta-illusion surface is set to 2 cm, and the transmitting horn antenna is placed approximately 2 m away from the test platform to ensure uniform plane-wave illumination across the device aperture. For comparative characterization, the reference virtual car and the meta-illusion device loaded with cuboid targets are sequentially placed at the identical position on the test platform, under illumination by a broadband x-polarized electromagnetic wave emitted from the horn antenna. The scanning probe, oriented parallel to the xoy plane, is driven by a three-dimensional near-field scanning system to map the full near-field electric field distributions, capturing both amplitude and phase information. The more detailed discussion is provided in Supplementary Note S2. Measured near-field results are summarized in Figure 7d–l. Figure 7d–f and Figure 5g–i present the near-field distributions of the standalone reference car and the target-loaded meta-illusion device, respectively, across 19, 21, and 23 GHz. The field patterns at all three representative frequencies exhibit high visual consistency, with only minor deviations attributed to inevitable fabrication tolerances and measurement uncertainties. To quantify the matching fidelity, one-dimensional electric field amplitude profiles extracted at z=80 mm are plotted in Figure 7j–l. The corresponding cosine similarity coefficients reach 0.90, 0.86, and 0.88 at 19, 21, and 23 GHz, respectively.

Consistent results from both far-field and near-field characterizations jointly verify that the proposed meta-illusion device achieves effective suppression of the real target’s intrinsic scattering signatures while accurately reconstructing the desired electromagnetic response under reflective operation, providing robust experimental evidence for the proposed meta-illusion functionality.

It should be noted that the electromagnetic response of the proposed meta-atom relies on the controlled coupling among multiple patterned metallic layers. Therefore, lateral misalignment between layers, deviations in dielectric and bonding-layer thicknesses, unintended air gaps, substrate-permittivity variations, conductor-patterning errors, and sample warpage may shift the resonances and perturb the designed phase dispersion. In the measurements, additional uncertainties may arise from antenna and sample alignment, polarization impurity, phase-reference drift over the broad frequency range, finite-aperture diffraction, residual scattering from the sample holder and chamber, and deviations from ideal plane-wave illumination. These factors are also responsible for part of the discrepancies between the measured responses of the meta-illusion device and the corresponding targets.

## 3. Conclusions

In conclusion, we propose and experimentally demonstrate a metasurface-based meta-illusion device capable of simultaneous target scattering suppression and virtual signature reconstruction, advancing conventional electromagnetic illusion technology from single-function free-space field synthesis to a dual-mode meta-illusion paradigm that integrates real-target concealment and virtual electromagnetic rendering. Distinct from previous schemes that focus exclusively on generating virtual scattering signatures in empty space, the proposed design framework directly modifies the electromagnetic scattering characteristics of a physically present target, transforming its response toward that of a predefined virtual object. This unified metasurface design conceals the inherent scattering of the real object while accurately synthesizing the desired virtual scattering distribution, offering a robust solution to the strongly coupled, highly nonlinear inverse problem arising from target-device electromagnetic interactions. The performance of the proposed strategy is thoroughly validated via both full-wave numerical simulations and microwave experimental measurements. High-fidelity scattering field reproduction is achieved across both near-field and far-field regions, with correlation coefficients exceeding 86% and 85% respectively, corroborating the excellent illusion fidelity of the device. It should be noted that the current demonstration is mainly focused on a single-source illumination condition with a specific polarization state. Extending the proposed meta-illusion strategy toward more complex electromagnetic environments, including multiple sources, multiple angles, multiple polarizations, and dynamic targets, will be an important direction for future research. This work establishes a scalable design framework for meta-illusion, opening new avenues for advanced intelligent electromagnetic camouflage, adaptive wavefront engineering, and reconfigurable electromagnetic sensing systems.

## Data Availability

The data that support the findings of this study are available from the corresponding author upon reasonable request.

## Supplementary Materials

The following supporting information can be downloaded at: https://www.mdpi.com/article/10.3390/mi17101146/s1, Figure S1: Schematic framework of the proposed Evolutionary Optimization Algorithm (EOA) for the inverse design of the meta-illusion device; Figure S2: Near field scanning measurement setup; Figure S3: Far field scanning measurement setup; Figure S4: Continuous frequency-sweep experimental verification of the broadband meta-illusion performance; Table S1: Comparison with representative metasurface-based electromagnetic illusion works [21,36,38,49].

## Abbreviations

The following abbreviations are used in this manuscript:

EOAs	Evolutionary Optimization Algorithms
PEC	Perfect Electric Conductor
PCB	Printed Circuit Board
VNA	Vector Network Analyzer
