# Supplementary material for: Ultrathin Metasurface-Enabled Electromagnetic Illusion for Target Platforms
Source: Micromachines (Basel). 2026 Sep 30;17(10):1146. doi: 10.3390/mi17101146 (PMC13707092; doi:10.3390/mi17101146)
Supplement: Supplementary file 1 [file micromachines-17-01146-s001.zip › micromachines-4585054-supplementary.pdf]

Supplementary Materials for

Ultrathin Metasurface-Enabled Electromagnetic Illusion for Target Platforms

The Supplementary Materials includes:

Supplementary Note S1: Detailed performance of the Evolutionary Optimization Algorithm

Supplementary Note S2: Experiment setup of the meta-illusion device

Supplementary Note S3. Continuous frequency-sweep measurement of the meta-illusion performance

Supplementary Note S4. Comparisons with representative metasurface-based electromagnetic illusion works

Supplementary Note S1. Detailed performance of the Evolutionary Optimization Algorithm

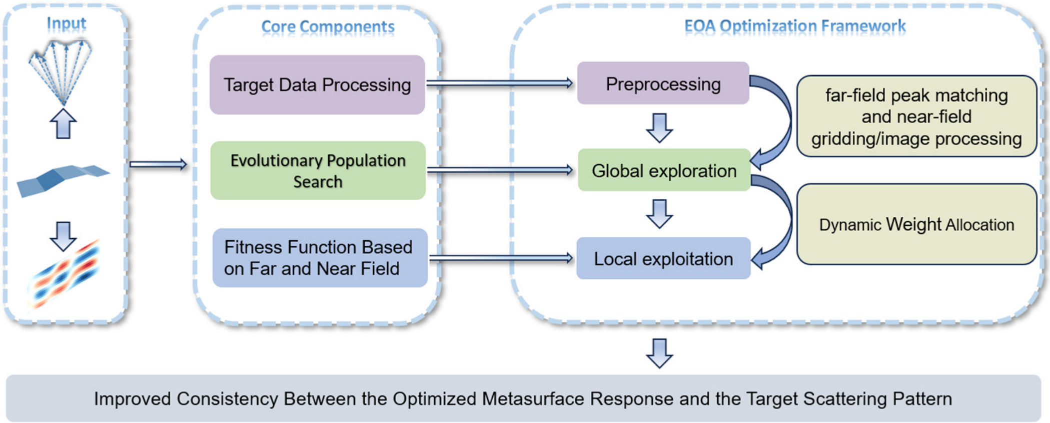

Figure S1 | Schematic framework of the proposed Evolutionary Optimization Algorithm (EOA) for the inverse design of the meta-illusion device. The algorithm takes three sets of physical parameters as inputs: the target far-field scattering pattern, metasurface phase response constraints, and the target near-field electric field distribution. Its architecture consists of three functional parts: target data processing, evolutionary population search, and a fitness function jointly constrained by far-field and near-field responses. Within the EOA optimization framework, a three-stage workflow of preprocessing, global exploration and local exploitation is implemented. A module of far-field peak matching and near-field gridding/image processing is integrated to assist the global exploration stage, while a dynamic weight allocation strategy is introduced to coordinate the local exploitation stage, balancing the matching fidelity of both far-field and near-field characteristics. The overall optimization goal is to achieve high consistency between the optimized metasurface scattering response and the predefined target scattering pattern, which forms the algorithmic foundation for the meta-illusion design presented in the main text.

The metasurface phase design problem addressed in this work can be formulated as a multi-constrained inverse scattering problem, where the phase profile of the metasurface is collaboratively optimized via a multi-objective joint optimization framework to achieve the target electromagnetic object substitution functionality. The core fitness function is constructed by integrating both far-field and near-field optimization objectives, expressed as:

$$F(\phi) = \omega_f[\alpha S_{dB} + \beta S_{lin} + \gamma S_{radio}] + \omega_n S_{near} - P(\phi)$$

where $\phi \in R^{N_x}$ denotes the phase distribution matrix to be optimized across the metasurface aperture, N_x is the total number of meta-atoms, ω_f and ω_n are weighting coefficients for the far-field and near-field contributions, and

$\omega_f + \omega_n = 1$. α, β and γ are the corresponding weighting coefficients in the far-field. S_{dB} denotes the peak-weighted cosine similarity of the far-field pattern in the decibel scale, defined as:

$$S_{\mathrm{dB}} = \frac{\sum_{k=1}^{k} w_k T_{dB}(k) O_{dB}(k)}{\left\| W\, T_{dB} \right\| \cdot \left\| W\, O_{dB} \right\|}$$

where T_{dB} and O_{dB} denote the far-field values of the target field and the optimized field, respectively. w_k is the peak attenuation weight defined as $w_k = 1 - 0.1(k-1)$, and W is a diagonal weighting matrix constructed from w_k (corresponding to the k-th peak). $\mathrm{S_{lin}}$ represents the linear-field intensity similarity, while S_{radio} denotes the multi-peak relative intensity matching metric. It is defined as:

$$S_{radio} = \frac{1}{1 + 10 \cdot MSE\left(\frac{T_{lin}^{(2:K)}}{T_{lin}^{(1)}}, \frac{O_{lin}^{(2:K)}}{O_{lin}^{(1)}}\right)}$$

S_{near} denotes the real-part similarity in the near field, defined based on the normalized complex electric field.

$$S_{near} = \frac{\Re(E_{opt} / \max\left|E_{opt}\right|) \cdot \Re(E_{tgt})}{\left\| \Re(E_{opt}) \right\| \cdot \left\| \Re(E_{tgt}) \right\|}$$

It should be noted that the purpose of this metric is to quantify the similarity between the optimized near-field distribution and the desired target near-field distribution in terms of their real components. The core idea is to compare the shape or mode profile of the two fields, rather than their absolute amplitudes. The resulting value is a scalar bounded between -1 and 1. $P(\phi)$ represents the phase constraint penalty term.

The proposed EOA framework adopts an iterative evolutionary optimization mechanism, with its workflow detailed as follows. First, a tournament selection strategy is employed to identify high-quality individuals. Then, uniform crossover and adaptive mutation operations are applied to enhance population diversity. To promote a balance between global exploration and local exploitation, a periodic joint information exchange mechanism is introduced, enabling elite migration and knowledge sharing among subpopulations. In addition, a dynamic weight adjustment strategy is incorporated to adaptively balance the contributions of different optimization objectives. The above process is executed iteratively until either the maximum number of iterations is reached or the solution converges to a predefined quality threshold. Once either of these termination conditions is satisfied, the optimization procedure is automatically stopped.

Supplementary Note S2. Experiment setup of the meta-illusion device

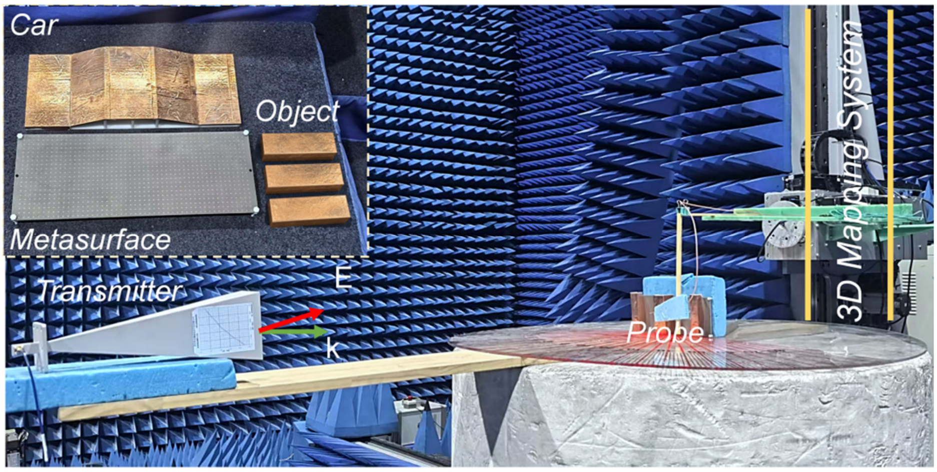

Figure S2 | Near field scanning measurement setup

The reference car model used in the experiment is fabricated via 3D printing, with a footprint dimension of 100mm×260mm. The three cuboid target objects share an identical width of 20mm, with heights set to 25mm, 27mm, and 25mm, respectively. All 3D-printed samples are used as physical reference targets for comparative measurements in both near-field and far-field experiments. The configuration of the near-field scanning measurement system is schematically illustrated in Figure S2. A transmitting horn antenna and a near-field scanning probe are connected to the VNA. The minimum distance between the meta-illusion prototype and the scanning probe is set to 2cm to suppress parasitic near-field coupling, and the transmitting horn antenna is placed approximately 2m away from the test platform to ensure uniform plane-wave illumination across the entire sample aperture. For the control-variable comparative measurement, the meta-illusion prototype and the 3D-printed reference target are sequentially placed at the identical position on the test platform, and illuminated by broadband x-polarized electromagnetic waves emitted from the transmitting horn antenna. The scanning probe, oriented parallel to the xoy plane, is driven by a three-dimensional near-field scanning system to perform a raster scan over a rectangular area of 300mm×120mm, capturing both amplitude and phase information of the near-field electric field.

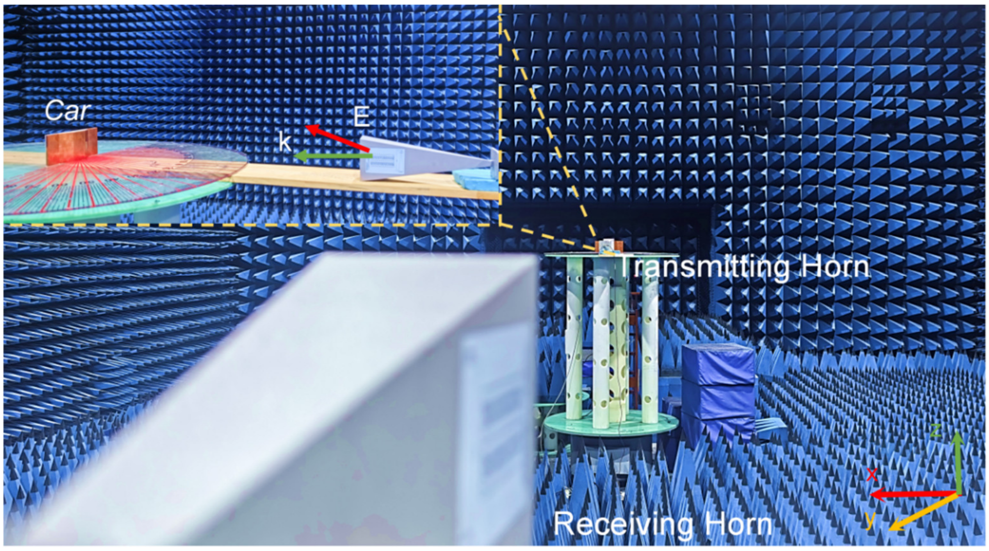

Figure S3 | Far field scanning measurement setup

The experimental setup for far-field scattering patterns are illustrated in Figure S3. The transmitting horn antennas and receiving horn antenna were connected to a vector network analyzer (VNA), which served as the microwave signal sources and receiver for scattering measurements. The VNA was calibrated using its standard automatic calibration procedure to remove systematic errors associated with the measurement chain. A power amplifier was employed in the transmitting path and a low-noise amplifier was used in the receiving path to improve the signal to noise ratio and reduce the influence of environmental electromagnetic interference. In addition, all measurements were carried out in a microwave anechoic chamber, where unwanted background reflections and ambient electromagnetic interference were substantially suppressed. The prototype was mounted on a 360° rotating platform to enable far-field pattern acquisition. Measurements were performed across the 18-26 GHz operating band, with the receiving antenna acquiring scattered field signals at each rotational step of the platform. The prototype was positioned at a distance of approximately 2 m from the transmitting antennas and 10 m from the receiving antenna, fully satisfying the far-field measurement conditions within the target operating band.

It should be noted that for the transmitting side, the purpose of placing the horn antenna sufficiently far from the metasurface is to ensure approximately plane-wave illumination over the sample aperture. Therefore, the relevant Fraunhofer criterion is determined by the aperture size of the transmitting horn antenna. The maximum aperture dimension of the horn antenna used in our experiment is approximately 100 mm. At the highest operating frequency of 23 GHz, the free-space wavelength is approximately 13.0 mm. According to the Fraunhofer criterion,$R_{FF} = 2D^2/\lambda$, the corresponding far-field distance is approximately 1.54 m.

Supplementary Note S3. Continuous frequency-sweep measurement of the meta-illusion performance

To further verify the broadband operating capability of the proposed meta-illusion device, continuous frequency-sweep measurements are conducted across the entire operating band of 19-23 GHz. The correlation coefficients between the measured electromagnetic responses of the meta-illusion device and the reference virtual target are calculated at different frequencies. As shown in Figure S4, both near-field and far-field correlations remain relatively

stable over the whole frequency range, confirming that the proposed device can maintain consistent electromagnetic illusion performance beyond the three representative frequencies presented in the main text.

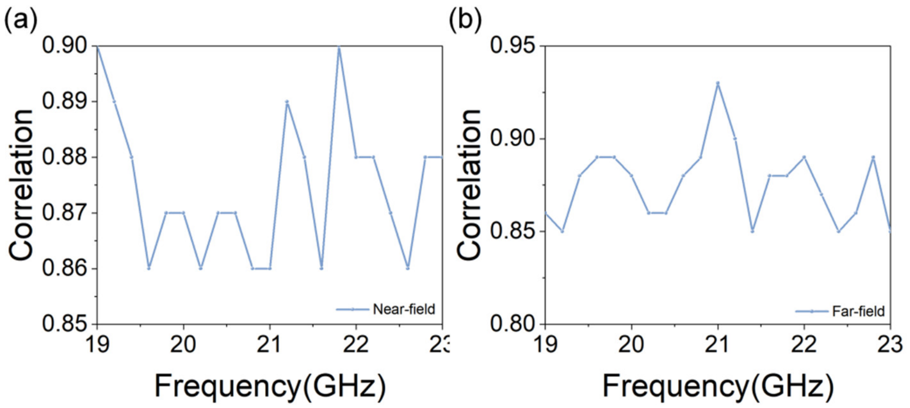

Figure S4. Continuous frequency-sweep experimental verification of the broadband meta-illusion performance. (a) Frequency-dependent correlation coefficients between the measured near-field distributions of the meta-illusion device and the reference virtual target over 19-23 GHz. (b) Frequency-dependent correlation coefficients between the measured far-field scattering patterns of the meta-illusion device and the reference virtual target over 19-23 GHz.

Supplementary Note S4. Comparisons with Representative Metasurface-Based Electromagnetic Illusion Works

Comparisons with previously reported electromagnetic-illusion metasurfaces in terms of operating band, polarization, operation mode, experimental validation, and field-pattern similarity are summarized in Table S1.

Table S1. Comparison with representative metasurface-based electromagnetic illusion works.

Ref.	Working Band (GHz)/bandwidth	Polarization	Mode	Experimental validation	Similarity
36	8-8.2 (2.4%)	Linear	Illusion	Near and far fields	72%-79%
21	6.8–7.6 GHz (11.1%) 12.9–13.7 GHz (6.0%)	Linear	Illusion	Near and Far fields	Near field: 77%-83%/75-81% Far field: 83-94%/79-82%
49	9–14 GHz (43.5%)	Linear	Illusion	Near field	/
38	12–18 GHz (40%)	Linear	Illusion	Far field	90.9%–98.4%
This Work	19–23 GHz (19.1%)	Linear	Illusion-stealth integrated	Near and far fields	Near field: 86%-90% Far field: 85%-93%
